# Mathematical Modeling and Optimizing of *in Vitro* Hormonal Combination for G × N15 Vegetative Rootstock Proliferation Using Artificial Neural Network-Genetic Algorithm (ANN-GA)

**DOI:** 10.3389/fpls.2017.01853

**Published:** 2017-11-01

**Authors:** Mohammad M. Arab, Abbas Yadollahi, Hamed Ahmadi, Maliheh Eftekhari, Masoud Maleki

**Affiliations:** ^1^Department of Horticultural Science, Faculty of Agriculture, Tarbiat Modares University, Tehran, Iran; ^2^Department of Horticulture, College of Aburaihan, University of Tehran, Tehran, Iran; ^3^Bioscience and Agriculture Modeling Research Unit, Department of Poultry Science, Tarbiat Modares University, Tehran, Iran

**Keywords:** artificial neural network (ANN), genetic algorithm (GA), G × N15 rootstock, cytokinin–auxin combination, proliferation, *Prunus* micro-propagation

## Abstract

The efficiency of a hybrid systems method which combined artificial neural networks (ANNs) as a modeling tool and genetic algorithms (GAs) as an optimizing method for input variables used in ANN modeling was assessed. Hence, as a new technique, it was applied for the prediction and optimization of the plant hormones concentrations and combinations for *in vitro* proliferation of Garnem (G × N15) rootstock as a case study. Optimizing hormones combination was surveyed by modeling the effects of various concentrations of cytokinin–auxin, i.e., BAP, KIN, TDZ, IBA, and NAA combinations (inputs) on four growth parameters (outputs), i.e., micro-shoots number per explant, length of micro-shoots, developed callus weight (CW) and the quality index (QI) of plantlets. Calculation of statistical values such as R^2^ (coefficient of determination) related to the accuracy of ANN-GA models showed a considerably higher prediction accuracy for ANN models, i.e., micro-shoots number: *R*^2^ = 0.81, length of micro-shoots: *R*^2^ = 0.87, CW: *R*^2^ = 0.88, QI: *R*^2^ = 0.87. According to the results, among the input variables, BAP (19.3), KIN (9.64), and IBA (2.63) showed the highest values of variable sensitivity ratio for proliferation rate. The GA showed that media containing 1.02 mg/l BAP in combination with 0.098 mg/l IBA could lead to the optimal proliferation rate (10.53) for G × N15 rootstock. Another objective of the present study was to compare the performance of predicted and optimized cytokinin–auxin combination with the best optimized obtained concentrations of our other experiments. Considering three growth parameters (length of micro-shoots, micro-shoots number, and proliferation rate), the last treatment was found to be superior to the rest of treatments for G × N15 rootstock *in vitro* multiplication. Very little difference between the ANN predicted and experimental data confirmed high capability of ANN-GA method in predicting new optimized protocols for plant *in vitro* propagation.

## Introduction

Artificial neural network and GA are among the most powerful calculation methods. ANN has developed as a very powerful and useful technique to model complicated non-linear systems (Woll and Cooper, 1997; Cook et al., 2000; Sadeghi, 2000; Chen and Ramaswamy, 2002; Chow et al., 2002; Arab et al., 2016; Jamshidi et al., 2016). However, finding the optimized amount of inputs combination in the case that we have different types and levels of each input to achieve the highest output is a problem. The GA optimization method can be applied as a famous technique to solve this problem (Cook et al., 2000; Chow et al., 2002).

So, a combined ANN-GA procedure was applied here to model and optimize the molding process of the *in vitro* plant hormones combination for the G × N15 *Prunus* rootstock. G × N15 is resistant to iron chlorosis (De la Guardia et al., 1995), drought, salinity and root-knot nematode (Meloidogyne) and suitable for *in vitro* propagation (Pinochet et al., 1999).

The effectiveness of ANNs has been pointed out in modeling and optimizing plant *in vitro* culture procedures (Gago et al., 2010b). ANNs have been successfully applied for modeling and optimizing estimation processes of the most appropriate *in vitro* culture medium for kiwifruit in which two variables of sucrose and light were modeled on kiwifruit shoot proliferation (Gago et al., 2014). As well, Gago et al. (2010a) modeled input variables of grape cultivar, time of exposure and concentration of IBA, on the *in vitro* rooting and acclimatization. Input variables of apricot varieties, different concentrations of BAP, K^+^, ${\mathrm{NO}}_{3}^{–}$, ${\mathrm{NH}}_{4}^{+}$, Ca^2+^, Cl^-^, Mg^2+^, ${\mathrm{PO}}_{4}^{2–}$, and ${\mathrm{SO}}_{4}^{2–}$ were modeled on output data of NS, LS and yield (NS × LS) and it was detected that ${\mathrm{NH}}_{4}^{+}$ is the main factor in shoot growth (Gago et al., 2011). In order to evaluate the benefits of ANN compared to classical statistical methods, an experiment was performed to find the effects of light intensity and sucrose amount on shoot number and length in proliferation stage (Gago et al., 2014).

In our similar works (Arab et al., 2016; Jamshidi et al., 2016) using ANN-GA hybrid method to predict and optimize the nutrients of the proliferation media for G × N15, Pyrodwarf and OHF rootstocks and comparing the mentioned method with regression modeling, the ANN-GA hybrid modeling was recognized as a high efficient and reliable method.

The type of hormones to use in the *in vitro* medium is important to achieve an efficient micropropagation process (Werbrouck, 2010; Amoo et al., 2011). BAP is crucial in plant tissue culture process as it has been used along with suitable auxins in micro-propagation of system (Ruzic and Vujovic, 2008). Furthermore, *in vitro* shooting is directly reliant on the axillary buds initiation and activity that are under the control of cytokinin hormones (Dobránszki and Teixeira da Silva, 2010). BAP, kinetin, zeatin, and thidiazuron have been exploited in combination with IBA and NAA for *in vitro* proliferation of *Prunus* rootstocks (Mante et al., 1989; Dobránszki and Teixeira da Silva, 2010; Arab et al., 2014) among which BAP and TDZ have been successfully used to induce regeneration of adventitious shoots (Ainsley et al., 2001) and it has been considerably affected by the auxin type and concentration. Accordingly, IBA and NAA could improve adventitious bud development in almond (Ainsley et al., 2001).

In the present study, we tried to optimize the *in vitro* medium hormones by using non-linear ANN-GA modeling and optimization procedure. We linked the model to GA to find the highest efficiency and the optimum concentrations of components which are critical for significant *in vitro* growth process. The objective of the this study was to model and optimize the appropriate *in vitro* hormone combination for proliferation of G × N15 rootstock and to assess the performance of the predicted and optimized cytokinin–auxin combination compared to the best found optimized concentrations in our other experiments.

## Materials and Methods

ANN-GA modeling and optimization procedure was used to construct optimized models by using the combination of different concentrations and types hormones as inputs and different measured *in vitro* growth parameters as outputs. The experiment details and description of the used technique to achieve the optimized inputs combinations are as follows.

## Case Study and Data

Sampling was carried out during April, May, June, and July from 15 to 20 cm new emerged shoots of G × N15 grown greenhouse. After transferring to the lab, shoots were cut to 1.5–3 cm single nodes. Then, explants were place 15–20 min into the solution of liquid detergent and water and were washed for 30–40 min under running tap water. Afterward, explants were disinfected in 0.2% benomyl and were rinsed with sterile distilled water. Subsequently, they were placed in 70% ethanol and were rinsed at least two times with sterile distilled water. And then explants were submerged for 4 min in mercury chloride (0.01%), with continuous movement. Then, the explants were immersed in sterile distillated water containing citric acid (700 mg/l) twice, each time 3 min and finally they were rinsed at least two times using sterile distilled water, before transfer to 200 ml jam jars containing 15 mL of culture medium supplemented with 0.25 mg/l BAP, 0.05 mg/l IBA. Originated shoots were sub-cultured on the same MS medium but supplemented with 1 mg/l BAP.

Murashige and Skoog (1962) (MS) basal medium having 30 g/l sucrose and 7 g/l agar with the pH of 5.8 was used in all experiments. Cultures of all experiments were incubated in a 16/8-h (light/dark) photoperiod at the light intensity of 80 μmol m^-2^ s^-1^ provided by white fluorescent tubes in a phytotron with temperature of 24 ± 2°C for 4 weeks. Three times sub-cultured proliferated explants were inoculated on free hormone MS medium for 2 weeks and forth subculture explants were used for assessing the optimized hormone combination in proliferation stage. Features like NS, LS, CW, and QI were evaluated.

## Experimental Design

All experiments were performed as completely randomized design (CRD) with factorial arrangement and 5 (third, fourth, and fifth experiments) to 6 (first and second experiments) replications each containing four explants.

In order to assess the combined effects of cytokinins (TDZ, KIN, and BAP) and auxins (IBA and NAA) on *in vitro* proliferation of G × N15 vegetative rootstock, five experiments were performed as follows: (Obtained data were used for modeling and optimization by ANN-GA.)

(1) BAP and IBA combination effect on proliferation rate (PR) (number of regenerated shoots per explant) was evaluated as 0, 0.5, 1, 1.5, and 2 mg/l BAP along with 0, 0.05, 0.1, and 0.15 mg/l IBA (**Table 1**).

**Table 1:** Interaction of BAP and IBA different concentrations on *in vitro* proliferation of G × N15.

Media	BAP (mg/l)	IBA (mg/l)	NS	LS (cm)	CW (g)	QI
1	0	0	1.33 ± 0.21	2.53 ± 0.06	0.01 ± 0.01	4.83 ± 0.11
2	0	0.05	1.33 ± 0.21	2.12 ± 0.05	0.02 ± 0.008	4.83 ± 0.11
3	0	0.1	1.67 ± 0.16	2.18 ± 0.06	0.07 ± 0.02	4.92 ± 0.08
4	0	0.15	1.67 ± 0.16	1.98 ± 0.03	0.04 ± 0.01	5.00 ± 0.00
5	0.5	0	3.17 ± 0.66	1.87 ± 0.03	0.09 ± 0.05	4.58 ± 0.15
6	0.5	0.05	3.67 ± 0.71	2.32 ± 0.03	0.10 ± 0.01	4.33 ± 0.11
7	0.5	0.1	3.33 ± 0.56	1.86 ± 0.04	0.14 ± 0.03	4.17 ± 0.11
8	0.5	0.15	3.17 ± 0.48	2.38 ± 0.05	0.19 ± 0.02	4.17 ± 0.11
9	1	0	8.33 ± 0.21	2.82 ± 0.03	0.16 ± 0.02	3.83 ± 0.11
10	1	0.05	9.00 ± 0.26	3.03 ± 0.04	0.18 ± 0.01	3.66 ± 0.11
11	1	0.1	10.67 ± 0.21	2.63 ± 0.02	0.22 ± 0.03	3.41 ± 0.15
12	1	0.15	6.67 ± 0.33	2.18 ± 0.07	0.27 ± 0.04	3.25 ± 0.11
13	1.5	0	3.67 ± 0.42	1.92 ± 0.03	0.18 ± 0.04	3.08 ± 0.15
14	1.5	0.05	3.33 ± 0.33	2.12 ± 0.05	0.21 ± 0.05	2.83 ± 0.11
15	1.5	0.1	3.83 ± 0.70	2.45 ± 0.03	0.18 ± 0.03	2.75 ± 0.21
16	1.5	0.15	2.67 ± 0.33	2.04 ± 0.04	0.27 ± 0.04	2.58 ± 0.15
17	2	0	3.00 ± 0.77	1.90 ± 0.03	0.36 ± 0.09	2.17 ± 0.17
18	2	0.05	2.33 ± 0.33	1.75 ± 0.03	0.20 ± 0.02	1.83 ± 0.11
19	2	0.1	2.33 ± 0.49	2.68 ± 0.04	0.31 ± 0.04	1.42 ± 0.15
20	2	0.15	4.00 ± 1.13	1.75 ± 0.04	0.35 ± 0.04	1.08 ± 0.08

(2) BAP and NAA combination effect on PR was evaluated as 0, 0.5, 1, 1.5, and 2 mg/l BAP and 0, 0.05, 0.1, and 0.15 mg/l NAA (**Table 2**).

**Table 2:** Interaction of BAP and NAA different concentrations on *in vitro* proliferation of G × N15.

Media	BAP (mg/l)	NAA (mg/l)	NS	LS (cm)	CW (g)	QI
1	0	0	1.16 ± 0.16	2.67 ± 0.04	0.00 ± 0.00	4.83 ± 0.11
2	0	0.05	1.83 ± 0.16	2.13 ± 0.05	0.01 ± 0.009	4.83 ± 0.11
3	0	0.1	1.17 ± 0.17	2.13 ± 0.06	0.02 ± 0.01	4.91 ± 0.08
4	0	0.15	1.17 ± 0.17	1.95 ± 0.02	0.06 ± 0.009	5.00 ± 0.00
5	0.5	0	2.83 ± 0.40	1.96 ± 0.04	0.07 ± 0.014	4.58 ± 0.15
6	0.5	0.05	4.17 ± 0.40	2.12 ± 0.05	0.09 ± 0.02	4.42 ± 0.08
7	0.5	0.1	4.00 ± 0.58	2.08 ± 0.11	0.11 ± 0.03	4.42 ± 0.11
8	0.5	0.15	3.33 ± 0.42	1.99 ± 0.08	0.16 ± 0.02	4.08 ± 0.15
9	1	0	6.67 ± 0.33	2.02 ± 0.03	0.15 ± 0.01	4.00 ± 0.00
10	1	0.05	7.50 ± 0.22	1.94 ± 0.05	0.19 ± 0.01	3.92 ± 0.08
11	1	0.1	8.50 ± 0.22	1.91 ± 0.04	0.23 ± 0.009	3.58 ± 0.15
12	1	0.15	6.00 ± 0.37	1.77 ± 0.04	0.26 ± 0.01	3.50 ± 0.00
13	1.5	0	3.83 ± 0.40	1.80 ± 0.03	0.21 ± 0.01	3.42 ± 0.08
14	1.5	0.05	3.67 ± 0.33	1.80 ± 0.04	0.25 ± 0.01	3.12 ± 0.11
15	1.5	0.1	3.33 ± 0.50	1.81 ± 0.05	0.28 ± 0.01	3.00 ± 0.13
16	1.5	0.15	2.83 ± 0.31	1.74 ± 0.04	0.32 ± 0.006	2.75 ± 0.11
17	2	0	2.50 ± 0.43	1.74 ± 0.03	0.24 ± 0.007	2.50 ± 0.13
18	2	0.05	2.33 ± 0.33	1.64 ± 0.02	0.27 ± 0.006	2.33 ± 0.11
19	2	0.1	2.00 ± 0.37	1.60 ± 0.03	0.33 ± 0.01	2.17 ± 0.11
20	2	0.15	2.67 ± 0.33	1.55 ± 0.02	0.35 ± 0.01	1.92 ± 0.08

(3) KIN and IBA combination effect on PR was evaluated as 0, 0.5, 1, 1.5, and 2 mg/l KIN and 0.05, 0.1, and 0.15 mg/l IBA (**Table 3**).

**Table 3:** Interaction of KIN and IBA different concentrations on *in vitro* proliferation of G × N15.

Media	KIN (mg/l)	IBA (mg/l)	NS	LS (cm)	CW (g)	QI
1	0	0.05	1.00 ± 0.00	2.16 ± 0.09	0.02 ± 0.005	4.60 ± 0.19
2	0	0.1	1.00 ± 0.00	2.04 ± 0.06	0.03 ± 0.003	4.00 ± 0.22
3	0	0.15	1.00 ± 0.00	1.98 ± 0.12	0.02 ± 0.003	4.10 ± 0.19
4	0.5	0.05	2.20 ± 0.20	1.76 ± 0.07	0.12 ± 0.017	3.60 ± 0.19
5	0.5	0.1	3.00 ± 0.32	1.62 ± 0.06	0.18 ± 0.019	3.40 ± 0.19
6	0.5	0.15	3.60 ± 0.24	1.50 ± 0.05	0.31 ± 0.012	3.10 ± 0.19
7	1	0.05	5.60 ± 0.40	1.42 ± 0.04	0.35 ± 0.014	2.90 ± 0.19
8	1	0.1	5.00 ± 0.32	1.28 ± 0.04	0.39 ± 0.014	2.7 ± 0.12
9	1	0.15	5.60 ± 0.51	1.26 ± 0.05	0.44 ± 0.005	2.30 ± 0.20
10	1.5	0.05	7.00 ± 0.32	1.34 ± 0.05	0.45 ± 0.015	2.00 ± 0.22
11	1.5	0.1	4.80 ± 0.37	1.22 ± 0.04	0.47 ± 0.015	1.90 ± 0.19
12	1.5	0.15	3.40 ± 0.24	1.08 ± 0.05	0.50 ± 0.10	1.40 ± 0.19
13	2	0.05	3.00 ± 0.32	1.18 ± 0.06	0.50 ± 0.021	2.30 ± 0.70
14	2	0.1	2.40 ± 0.24	1.08 ± 0.04	0.51 ± 0.027	2.00 ± 0.76
15	2	0.15	1.80 ± 0.20	1.08 ± 0.04	0.63 ± 0.017	1.50 ± 0.63

(4) KIN and NAA combination effect on PR was evaluated as 0, 0.5, 1, 1.5, and 2 mg/l KIN and 0.05, 0.1, and 0.15 mg/l NAA (**Table 4**).

**Table 4:** Interaction of KIN and NAA different concentrations on *in vitro* proliferation of G × N15.

Media	KIN (mg/l)	NAA (mg/l)	NS	LS (cm)	CW (g)	QI
1	0	0.05	1.00 ± 0.00	2.06 ± 0.13	0.02 ± 0.02	5.00 ± 0.00
2	0	0.1	1.00 ± 0.00	2.14 ± 0.17	0.07 ± 0.12	4.70 ± 0.27
3	0	0.15	1.00 ± 0.00	2.46 ± 0.11	0.02 ± 0.01	4.60 ± 0.22
4	0.5	0.05	3.00 ± 0.71	1.90 ± 0.07	0.07 ± 0.03	4.30 ± 0.27
5	0.5	0.1	3.60 ± 1.14	2.28 ± 0.08	0.12 ± 0.05	4.00 ± 0.00
6	0.5	0.15	4.60 ± 1.14	2.38 ± 0.24	0.17 ± 0.06	4.00 ± 0.00
7	1	0.05	5.40 ± 0.89	1.92 ± 0.08	0.16 ± 0.02	3.60 ± 0.22
8	1	0.1	6.80 ± 0.84	1.74 ± 0.11	0.22 ± 0.03	3.50 ± 0.00
9	1	0.15	7.40 ± 0.89	1.66 ± 0.05	0.27 ± 0.03	3.20 ± 0.27
10	1.5	0.05	6.40 ± 0.55	1.76 ± 0.11	0.25 ± 0.03	3.00 ± 0.00
11	1.5	0.1	9.80 ± 0.84	1.52 ± 0.16	0.30 ± 0.04	2.7 ± 0.27
12	1.5	0.15	6.20 ± 0.84	1.64 ± 0.13	0.33 ± 0.03	2.50 ± 0.00
13	2	0.05	3.80 ± 0.45	1.52 ± 0.08	0.30 ± 0.05	2.20 ± 0.27
14	2	0.1	4.80 ± 1.09	1.28 ± 0.08	0.36 ± 0.03	2.00 ± 0.00
15	2	0.15	2.80 ± 0.45	1.32 ± 0.08	0.39 ± 0.02	1.30 ± 0.27

(5) TDZ and IBA combination effect on PR was evaluated as 0.5, 1, 1.5, and 2 mg/l TDZ and 0, 0.05, 0.1, and 0.15 mg/l IBA (**Table 5**).

**Table 5:** Interaction of TDZ and IBA different concentrations on *in vitro* proliferation of G × N15.

Media	TDZ (mg/l)	IBA (mg/l)	NS	LS (cm)	CW (g)	QI
1	0	0.05	1.00 ± 0.00	1.96 ± 0.02	0.04 ± 0.007	3.50 ± 0.00
2	0	0.1	1.00 ± 0.00	1.82 ± 0.04	0.05 ± 0.003	3.30 ± 0.12
3	0	0.15	1.00 ± 0.00	1.66 ± 0.02	0.04 ± 0.004	3.10 ± 0.10
4	0.5	0.05	1.40 ± 0.24	1.76 ± 0.02	0.21 ± 0.012	2.90 ± 0.10
5	0.5	0.1	2.00 ± 0.00	1.60 ± 0.03	0.39 ± 0.013	2.60 ± 0.10
6	0.5	0.15	3.00 ± 0.00	1.52 ± 0.02	0.48 ± 0.029	2.50 ± 0.00
7	1	0.05	2.60 ± 0.40	1.62 ± 0.04	0.38 ± 0.012	2.30 ± 0.12
8	1	0.1	3.80 ± 0.73	1.42 ± 0.04	0.44 ± 0.007	2.00 ± 0.00
9	1	0.15	2.80 ± 0.37	1.30 ± 0.03	0.56 ± 0.023	1.90 ± 0.10
10	1.5	0.05	2.80 ± 0.37	1.42 ± 0.04	0.41 ± 0.017	1.60 ± 0.10
11	1.5	0.1	5.00 ± 0.00	1.30 ± 0.03	0.47 ± 0.016	1.50 ± 0.00
12	1.5	0.15	4.4 ± 0.24	1.18 ± 0.02	0.53 ± 0.026	1.10 ± 0.10
13	2	0.05	4.20 ± 0.58	1.32 ± 0.04	0.41 ± 0.013	1.00 ± 0.00
14	2	0.1	3.60 ± 0.24	1.10 ± 0.03	0.55 ± 0.020	0.80 ± 0.12
15	2	0.15	2.20 ± 0.49	0.84 ± 0.05	0.70 ± 0.022	0.50 ± 0.00

## Modeling and Optimization of ANN-GA Hybrid System

### ANN Modeling

Artificial neural network is an appropriate mathematical structure including an inter-connected set of processing elements or nodes. The architecture of a simple ANN comprises three layers including input, output, and hidden layers. It has been shown in many literatures that a single hidden layer is enough for ANN for computing complex non-linear relationships. A mathematical function is used for processing the information of the input layer to transfer to the hidden layer. The input data are added using a propagation function a single response is generated as the out value in output layer. The output value will be compared to the experimental value and the error made by the ANN can be estimated (Suárez et al., 2015).

In this study, a common network algorithm comprising the feed forward back-propagation (3-layer back-propagation network) was used to make an ANN model. Transfer functions of hyperbolic tangent sigmoid (tansig) and linear (purelin) were applied for hidden and output layers, respectively. A Levenberg-Marquardt algorithm for back-propagation with a gradient descent and momentum weight and bias learning function was used for training of the network (Demuth et al., 2006). Performance function of mean square error (MSE) with 0.01 level was used and training was terminated after 800 epochs or iterations of the network. Units in the input layer of the ANN model included five input variables of IBA, NAA, KIN, BAP, and TDZ with different levels (**Tables 1–5**). Four models were constructed separately for NS, LS, CW, and QI. 279 and 186 data lines were used to train and test the network. The data sets of input and output were normalized (between -1 and 1) simplifying the problem for the network, in order to obtain fast conjunction minimum MSE, and to make sure that the recreation of targets (output data) fall into the new feed forward network specific range can be attained (Demuth et al., 2006; Gulati et al., 2010; Ahmadi and Golian, 2011). *R*^2^ (coefficient of determination) and RMSE (root mean square error), were used to evaluate the fitness of the ANN-model (Ahmadi et al., 2007) as follows:

(1) $$R^{2}\;=\;{\left(\frac{n(\sum xy)-(\sum x)(\sum y)}{\sqrt{\left[n\sum x^{2}-{\left(\sum x\right)}^{2}][n\sum y^{2}\;-\;{\left(\sum y\right)}^{2}\right]}}\right)}^{2}$$

(2) $$\text{RMSE}\;\text{=}\;\sqrt{(\sum_{\text{i=1}}^{\text{n}}{\left(\text{M-O}\right)}^{\text{2}})\text{/n}}$$

### Genetic Algorithm Optimization

The significance of GAs as strong tools for optimizing has been highly assessed with different uses (Wang, 2001). It is a kind of parallel iterative optimization algorithm with “generation-evaluation” and given learning capability, that repeats the steps of evaluation, selection, mutation, and crossover to satisfy the stopping condition. The individual or member in a population is referred by a chromosome comprising genes. The criterion value is associated to the individual for optimization. Then the populations of individuals are generated iteratively and the process of selection, crossover, and mutation are performed on it. The selection is done to promote the most appropriate members of the population for the determined criterion. Crossover and mutation ensure the probing of the state area (Idrissi et al., 2016).

To determine the optimum amounts and combinations of input variables of IBA, NAA, KIN, BAP, and TDZ to achieve the best values of outputs NS, LS, CW and QI, the trained ANN models were processed as the fitness function by GA.

**FIGURE 1:**
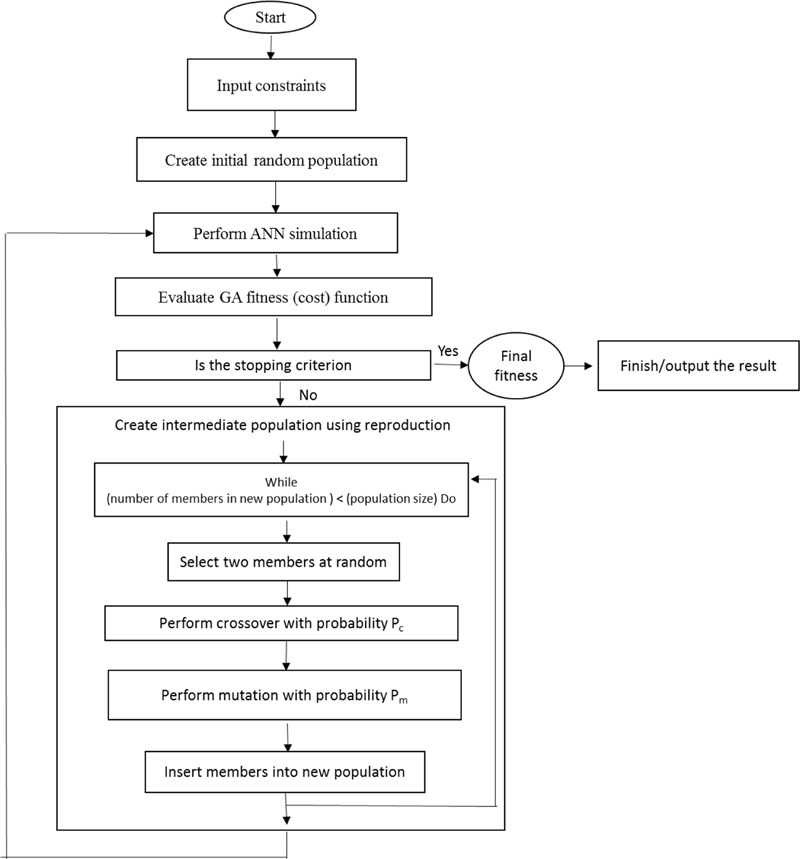
An overall block diagram of ANN-GA method used in the manuscript (adopted from Jamshidi et al., 2016).

In order to attain the best fitness, the selection method of roulette wheel was used to select elite populations for crossover. 50 initial populations, 500 generations number, 0.1 mutation rate, and 0.85 crossover rate were set (Haupt and Haupt, 1998; Demuth et al., 2006). To achieve the generations number, generational practice was performed repeatedly (**Figure 1**). The search for the optimal solutions was restricted between the input variable limits through performing GA, determined in the CRD.

### Ranking the Relative Importance of Input Variables

Sensitivity analysis process was implemented on the obtained ANN model to recognize the importance of input variables in the model. This analysis shows which IBA, NAA, BAP, KIN, and TDZ concentrations and combinations are more important than the other to achieve an optimal NS, SL, CW, and QI of G × N15 rootstock explants. The sensitivity of NS, SL, CW, and QI against the examining hormones was determined by the criteria (Lou and Nakai, 2001; Ahmadi and Golian, 2010a,b) including the variable sensitivity error (VSE) value which shows the performance of the constructed ANN-GA model when that variable is unavailable and VSR value which indicates the ratio of VSE and ANN model error if all variables are available. Higher VSR value indicates that the variable is more important. Accordingly, on the basis of the found VSR value, the input variables can be ranked according their importance (Ahmadi and Golian, 2010a,b).

Mathematical code was written using Matlab (Matlab, 2010) software for constructing and assessing the ANN-GA model. In fact, the established program is an altered source code previously used by Ahmadi and Golian (2011) for an ANN algorithm.

Here, the best combined concentrations of cytokinin–auxin hormones predicted and optimized by ANN-GA resulting in the highest PR were tested.

## Results

(1) BAP + IBA concentrations effect on proliferation
  The highest PR (10.67) was detected in 1 mg/l BAP + 0.1 mg/l IBA. As well, the highest LS (3.03 cm) was found in 1 mg/l BAP + 0.05 mg/l IBA. The control without hormone treatment resulted in the lowest CW and QI (**Table 1**).
(2) BAP + NAA concentrations effect on proliferation
  The highest PR (8.5) was obtained in 1 mg/l BAP + 0.1 mg/l NAA. The control without hormone treatment resulted in the highest LS (2.13 cm) (**Table 2**).
  The results of these two experiments showed that BAP + IBA hormonal combination is more efficient than BAP + NAA for *in vitro* proliferation of G × N15. On the other hand, it is evidence of the results that more concentration of KIN than BAP is to be used.
(3) KIN + IBA concentrations effect on proliferation
  The highest PR (7) was attained in the 1.5 mg/l KIN + 0.05 mg/l IBA. The highest LS was found in control without hormone treatment (**Table 3**).
(4) KIN + NAA concentrations effect on proliferation
  The highest PR (9.80) was detected in 1.5 mg/l KIN + 0.1 mg/l NAA (**Table 4**). KIN + NAA was found as a more efficient hormonal combination than KIN + IBA for *in vitro* proliferation of G × N15.
(5) TDZ + IBA concentrations effect on proliferation
  The highest PR (5) was acquired in 1.5 mg/l TDZ + 0.1 mg/l IBA (**Table 5**).

These results show that both the type and concentration cytokinin–auxin combination play critical role in proliferation and each plant species needs a special concentration of hormones according its internal hormones content.

## ANN-GA Modeling and Optimization, and Sensitivity Analysis

### ANN Modeling and Evaluation

**FIGURE 2:**
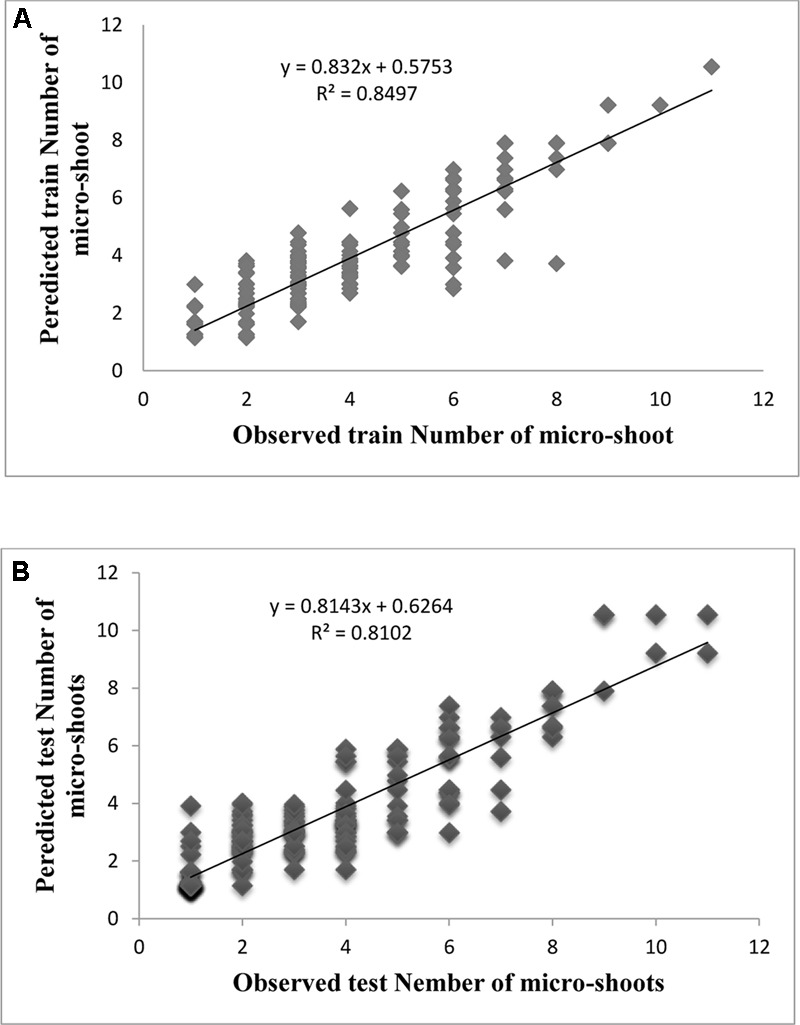
Observed vs. ANN-GA model-predicted values of NS: **(A)** training set (*n* = 279); **(B)** testing set (*n* = 186) for G × N15.

**FIGURE 3:**
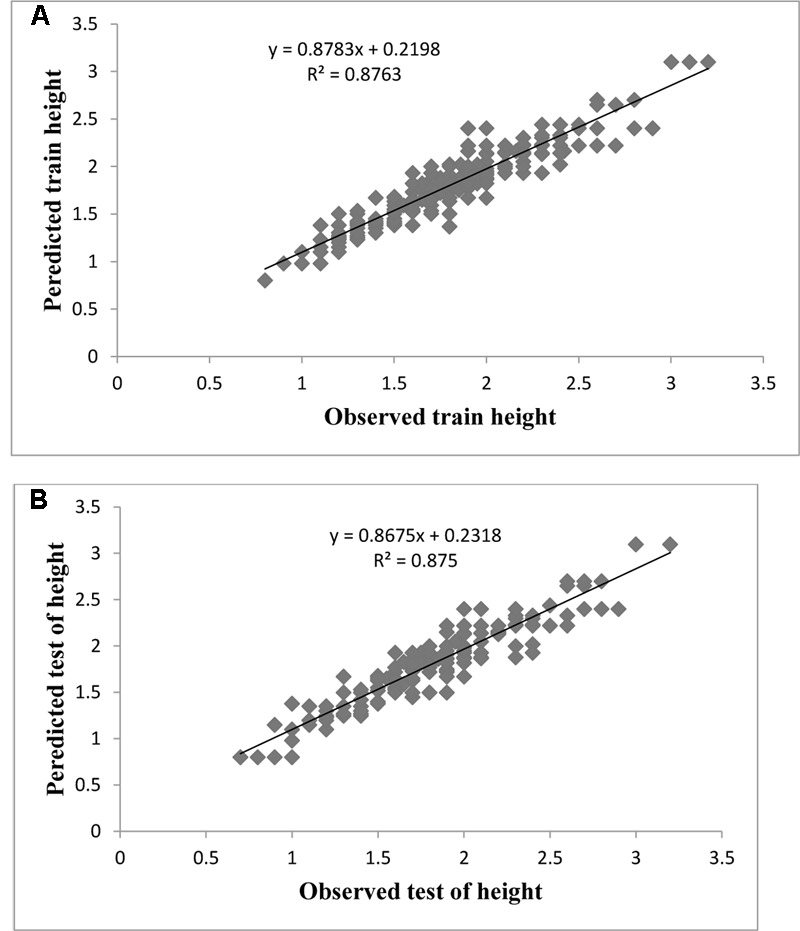
Observed vs. ANN-GA model-predicted values of LS: **(A)** training set (*n* = 279); **(B)** testing set (*n* = 186) for G × N15.

**FIGURE 4:**
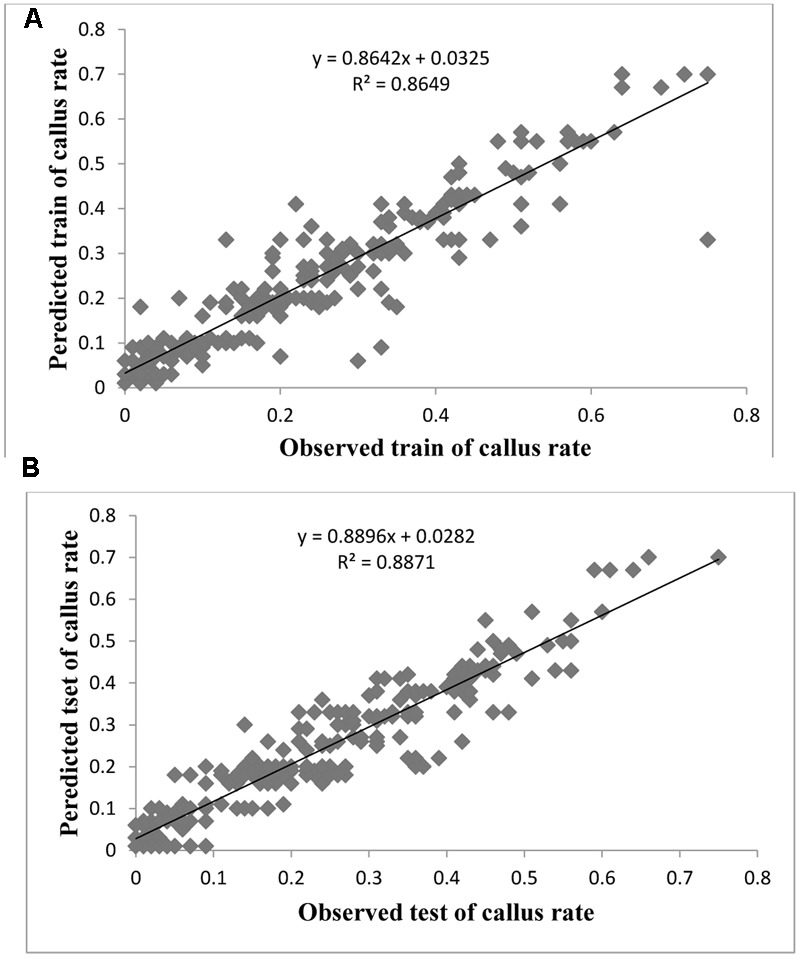
Observed vs. ANN-GA model-predicted values of CW: **(A)** training set (*n* = 279); **(B)** testing set (*n* = 186) for G × N15.

**FIGURE 5:**
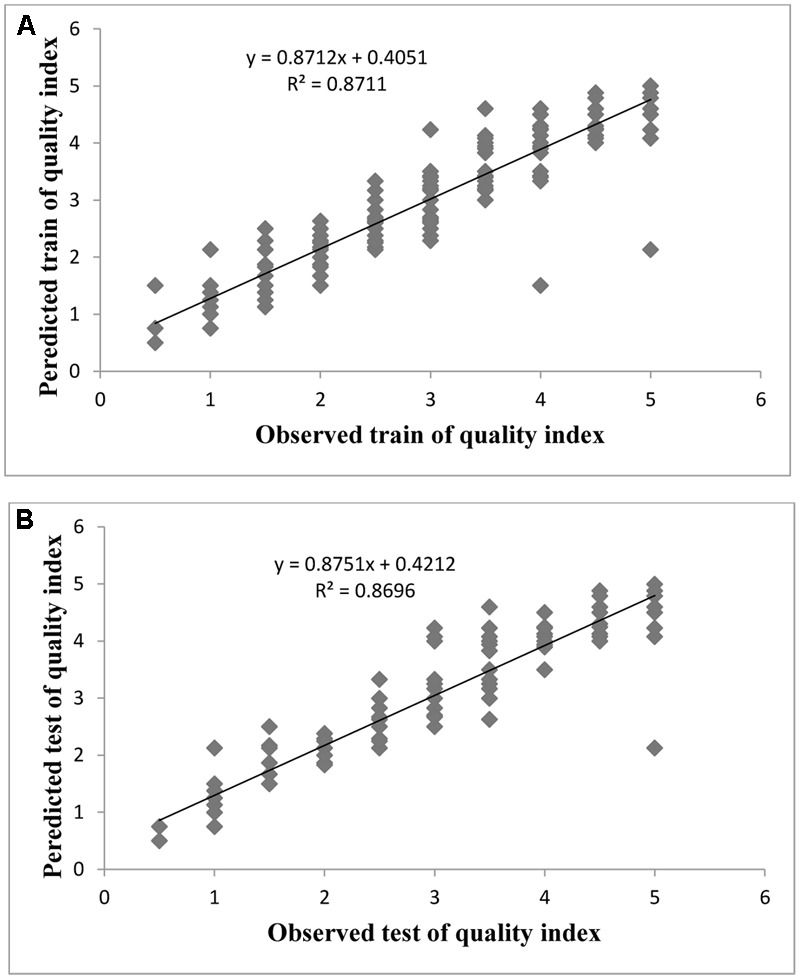
Observed vs. ANN-GA model-predicted values of QI: **(A)** training set (*n* = 279); **(B)** testing set (*n* = 186) for G × N15.

Evaluation of observed and predicted outputs defines the performance of the ANN-model of studying inputs. The results showed effective correspondence between the observed and the predicted values of explant growth parameters for both training and testing sets (**Table 6**). The fitted simple regression lines indicate high accordance between the observed and predicted values of NS, LS, CW, and QI for both the training and testing sets. Using high squared correlation coefficients fitting method and according to the ANN models obtained, eight graphs were created to display the variation in NS, LS, CW, and QI at different concentrations and combinations of hormones IBA, NAA, BAP, KIN, and TDZ (**Figures 2–5**). The graphs may be helpful to perceive the complete hormones response relationship and to assess the combined effects of various concentrations and combinations of hormones. The ANN models could accurately (*R*^2^ > 81, 87, 86, and 87) predict NS, LS, CW, and QI in the testing data sets that were not used throughout the training processes (**Figures 2–5**). Moreover, the trained ANN models of NS, LS, CW, and QI showed balanced statistical values for both subsets of training and testing (**Table 6**). Generally, statistical values (**Table 6**) revealed that the ANN-based models could effectively fit published data on the performances of G × N15 micro-shoots throughout *in vitro* multiplication to various concentrations and combinations of hormones.

**Table 6:** Statistics of ANN models for NS, LS, CW and QI of G × N15 (training vs. testing values).

	NS		LS		CW		QI	
Item	Training	Testing	Training	Testing	Training	Testing	Training	Testing
R Square	0.85	0.81	0.88	0.87	0.86	0.88	0.87	0.87
RMSE	0.83	1.02	0.15	0.15	4.53	0.05	0.43	0.45
*t*-Test	0.86	0.54	0.89	0.25	0.99	0.62	0.97	0.46

### Model Optimization and Sensitivity Analysis of the Models

#### The ANN-GA Predicted Optimized Amounts of Different Auxin–Cytokinin Combinations

**Table 7** shows the results of five experiments on different auxin–cytokinin combinations using ANN-GA to predict optimized values of shooting. Among different treatments, the highest optimized predicted shooting (10.53) obtained in 1.02 mg/l BAP + 0.098 mg/l IBA and the lowest one (4.98) attained in 1.5 mg/l TDZ + 0.052 mg/l IBA (**Table 7**). VSR results showed that BAP and KIN both had the highest effects and NAA had the lowest effect on *in vitro* shooting of G × N15 vegetative rootstock (**Table 7**).

**Table 7:** Importance of hormones concentrations (mg/l) and combination according to the sensitivity analysis and optimization analysis on the developed ANN-GA model to reach maximum *in vitro* proliferation rate in G × N15.

Item	Hormones concentrations (mg/l) (input variable)					Predicted proliferation rate (output variable) at optimal point
	BAP	IBA	NAA	KIN	TDZ	
BAP + IBA	1.02	0.098	0	0	0	10.53
BAP + NAA	0.98	0	0.1	0	0	7.89
KIN + IBA	0	0.01	0	1.46	0	6.62
KIN + IBA	0	0	0.048	1.53	0	9.19
TDZ + IBA	0	0.052	0	0	1.5	4.98
VSR	19.3	2.63	1.81	9.64	2.25	
Rank	1	3	5	2	4	

**Table 8** shows the predicted optimized results of cytokinin–auxin combinations to obtain the highest LS using ANN-GA. Among all defined hormonal combinations, the highest (3.1 cm) predicted optimized LS was predicted to be achieved with 1.08 mg/l BAP + 0.068 mg/l IBA and the lowest (2.3 cm) one was predicted to be in reaction of the hormonal combination of 0.23 mg/l TDZ + 0.02 mg/l IBA. The results of VSR analysis showed that BAP and KIN were respectively the highest effective hormones on LS while NAA had the lowest effect on it **Table 8**.

**Table 8:** Importance of hormones concentrations (mg/l) and combination according to the sensitivity analysis and optimization analysis on the developed ANN-GA model to reach maximum *in vitro* height in G × N15.

Item	Hormones concentrations (mg/l) (input variable)					Predicted height (output variable) at optimal point
	BAP	IBA	NAA	KIN	TDZ	
BAP + IBA	1.08	0.068	0	0	0	3.1
BAP + NAA	1.09	0	0.017	0	0	2.4
KIN + IBA	0	0.09	0	0.23	0	2.3
KIN + IBA	0	0	0.1	0.4	0	2.4
TDZ + IBA	0	0.02	0	0	0.23	2.3
VSR	12.9	4.7	3.1	6.8	6.4	
Rank	1	4	5	2	3	

**Table 9** shows the predicted optimized results of cytokinin–auxin combinations to obtain the lowest CW using ANN-GA. Results indicated that the lowest CW (0 g) was detected in the control without hormone medium. VSR analysis showed that TDZ, KIN, and BAP were respectively the highest (12.7, 7.7, and 4) and IBA and NAA were respectively the lowest (2.7 and 1.9 g) effective hormones on callus derived from end of new micro- shoots (**Table 9**).

**Table 9:** Importance of hormones concentrations (mg/l) and combination according to the sensitivity analysis and optimization analysis on the developed ANN-GA model to reach minimum *in vitro* CW in G × N15.

Item	Hormones concentrations (mg/l) (input variable)					Predicted callus weight (output variable) at optimal point
	BAP	IBA	NAA	KIN	TDZ	
BAP + IBA	0	0	0	0	0	0.007
BAP + NAA	0	0	0	0	0	0.007
KIN + IBA	0	0	0	0	0	0.007
KIN + IBA	0	0	0	0	0	0.007
TDZ + IBA	0	0	0	0	0	0.007
VSR	4	2.7	1.9	7.7	12.7	
Rank	3	4	5	2	1	

**Table 10** shows the predicted optimized results of cytokinin–auxin combinations to obtain the highest quality of shoot using ANN-GA. The highest (5) optimized shoot quality was predicted in all media containing low concentrations of cytokinin (**Table 10**). Since *in vitro* plants require an external cytokinin to proliferate, 0.65 mg/l KIN and 0.15 mg/l IBA may be an appropriate hormonal combination as it causes to produce high quality shoots in addition to high NS. VSR analysis showed that TDZ and KIN were the highest effective hormones and NAA was the lowest effective hormone on quality of micro-shoots (**Table 10**).

**Table 10:** Importance of hormones concentrations (mg/l) and combination according to the sensitivity analysis and optimization analysis on the developed ANN-GA model to reach maximum *in vitro* QI in G × N15.

Item	Hormones concentrations (mg/l) (input variable)					Predicted quality index (output variable) at optimal point
	BAP	IBA	NAA	KIN	TDZ	
BAP + IBA	0.13	0.14	0	0	0	5
BAP + NAA	0.17	0	0.14	0	0	5
KIN + IBA	0	0.15	0	0.65	0	5
KIN + IBA	0	0	0.13	0.38	0	5
TDZ + IBA	0	0.15	0	0	0.12	5
VSR	4.5	1.7	1.8	5.5	9.1	
Rank	3	5	4	2	1	

#### Optimized Values of Cytokinin–Auxin on Shooting Predicted by ANN-GA

Initially, we analyzed each level of cytokinin–auxin combination separately. The used medium was modified MS which was obtained in the primary experiments. Then, in order to find the optimized hormonal combination and concentration, all experiments data were analyzed simultaneously using ANN-GA and the optimized concentration of hormonal combination was predicted. So, 1.02 mg/l BAP + 0.098 mg/l IBA was predicted to result in the highest NS (10.53) and also 1.53 mg/l KIN + 0.048 mg/l NAA was predicted to be an appropriate (9.19) composition for the proliferation stage. Mixture of 0.98 mg/l BAP + 0.1 mg/l NAA (7.89) was realized as a less effective treatment than two previously mentioned combinations. Higher NS obtained in KIN and NAA toward BAP and NAA combination confirms that the interaction of cytokinin and auxin effects on shooting and choosing the appropriate type of cytokinin and auxin and their concentrations is critical for shooting. 1.08 mg/l BAP + 0.068 mg/l IBA (3.1 cm) and 0.4 mg/l KIN + 0.1 mg/l NAA (2.4 cm) were predicted as optimized hormonal combination for LS. The highest QI was possible to achieve but by decreasing cytokinin concentration but it will bring about shooting reduction. So, 0.65 mg/l KIN + 0.15 mg/l IBA is proposed as an appropriate hormonal combination for achieving high quality shoots.

In general, according to the ANN-GA analysis results on different parameters of *in vitro* proliferation, hormonal combination of BAP and IBA is predicted to be more proper than other combinations in *in vitro* multiplication of G × N15 rootstock due to producing higher number and LS. In accordance with the present study results, in the several number of studies BAP has been known as the best cytokinin for *in vitro* proliferation of *Prunus* genus and it has been used in combination with very low concentrations of auxins (Wagner, 2003) and also IBA has been known as the most famous used auxin for *in vitro* proliferation of *Prunus* (Silveira et al., 2002). Although the combination of KIN and NAA produced high number of qualified shoots but regenerated shoots were shorter and weaker than ones in BAP + IBA treatments. According to the above results, ANN-GA can be considered as one of the high efficient methods in analyzing data obtained of *in vitro* proliferation parameters for predicting optimized hormonal combination (type and concentration of cytokinin–auxin hormones) required in the proliferation stage.

#### Validation Experiment (Assessment of Optimum Productivity of New Media Formulated)

Interaction of cytokinin–auxin affected significantly (*p* < 0.001) on PR, LS, CW, and QI. So that the highest PR (10.80) was detected in 1 mg/l BAP + 0.1 mg/l IBA (**Table 11**). The highest LS (2.90 cm) was found in 1.5 mg/l BAP + 0.1 mg/l IBA (**Table 11**). And the highest CW (0.46 g) was recorded in the 1.5 mg/l KIN + 0.052 mg/l IBA (**Table 11**). Our results showed that enhancing hormones concentrations caused CW increase and LS decrease. The highest QI (4.01) was obtained in 1.46 mg/l KIN + 0.01 mg/l IBA (**Table 11**). Totally, it can be concluded that among different hormones, BAP and IBA play efficient role in *in vitro* proliferation of G × N15 and the weak effect of KIN may be due to its lower cytokinin power. As well, our results show that interaction of hormones is important in *in vitro* proliferation of G × N15 so that BAP + IBA and KIN + NAA were effective on G × N15 *in vitro* proliferation. The results of verification analysis showed that the combination of ANN-GA is an efficient method for prediction and optimization of hormones combination in *in vitro* proliferation, so that the optimized predicted combination of 1/2 mg/l BAP + 0.098 IBA was found the best by ANN-GA achieving 10.20 NS which although a little bit lower than one predicted about the treatment of 1 mg/l BAP + 0.1 mg/l IBA but a higher QI was attained with the first combination.

**Table 11:** Effect of different concentrations and combinations of hormones on *in vitro* growth parameters.

Hormones combination	Proliferation	LS (cm)	CW (g)	QI
1 mg/l BAP + 0.1 mg/l IBA	10.80 ± 0.37 a	2.90 ± 0.03 a	0.17 ± 0.008 e	3.65 ± 0.06 d
1 mg/l BAP+ 0.1 mg/l NAA	8.60 ± 0.24 cd	2.30 ± 0.03 c	0.24 ± 0.021 d	3.85 ± 0.06 cd
1.5 mg/l KIN + 0.05 mg/l IBA	7.00 ± 0.32 e	1.90 ± 0.01 e	0.41 ± 0.010 b	4.20 ± 0.05 ab
1.5 mg/l KIN + 0.05 mg/l NAA	9.60 ± 0.24 abc	2.05 ± 0.03 d	0.32 ± 0.008 c	3.95 ± 0.05 bcd
1.5 mg/l KIN + 0.1 mg/l IBA	5.00 ± 0.32 f	1.60 ± 0.02 f	0.42 ± 0.011 ab	2.50 ± 0.08 e
1.2 mg/l BAP + 0.098 mg/l IBA	10.20 ± 0.37 ab	2.82 ± 0.02 a	0.19 ± 0.005 de	3.85 ± 0.10 cd
0.98 mg/l BAP + 0.1 mg/l NAA	7.60 ± 0.24 de	2.50 ± 0.04 b	0.24 ± 0.006 d	4.00 ± 0.08 bc
1.46 mg/l KIN + 0.01 mg/l IBA	6.80 ± 0.20 e	2.36 ± 0.02 bc	0.38 ± 0.007 b	4.35 ± 0.06 a
1.53 mg/l KIN + 0.048 mg/l NAA	9.40 ± 0.24 bc	2.40 ± 0.04 bc	0.33 ± 0.009 c	4.10 ± 0.06 abc
1.5 mg/l KIN + 0.052 mg/l IBA	4.40 ± 0.24 f	1.84 ± 0.04 e	0.46 ± 0.008 a	2.35 ± 0.06 e
*p-*Value				
Medium	<0.001	<0.001	<0.001	<0.001

*Values in each column represent means ± SE. Different letters within a column indicate significant differences (*p* < 0.05).*

## Discussion

The success in micro-propagation and the commercial value of plant micro-propagation protocols such as fruit tree rootstocks of *Prunus* genus is extensively related to the PR. As there are rare studies on micro-propagation of G × N15 vegetative rootstock, we evaluated the combined effects of different cytokinins of BAP, KIN, and TDZ with auxins of IBA and NAA after determining optimized culture medium.

Plant hormonal interactions create a crucial intricacy in the regulation of developmental processes by offering their two complementary aspects namely robustness and stability as well as dynamicity and flexibility. Newly increased understandings of the molecular mechanisms underlying the hormonal systems obviously reveal their developmental role (Vanstraelen and Benková, 2012). The balance between cytokinin and auxin is critical for the development and preservation of meristems. Essentially, hormonal systems have a robust flexibility and make it possible for input to integrate several internal and external signals that influence different developmental processes during the plant growth by multiple transcriptional and post-transcriptional interactions involved in metabolism, transport, hormone signaling, and downstream reactions. A nearby insight of the elementary regulatory chains shows the presence of common interaction units that are adopted using various hormonal systems irrespective of the developmental background (Vanstraelen and Benková, 2012).

Cytokinin plays a key role in forming the organization and regulating cell division in the shoot apical meristem, as it has been shown that cytokinin regulates cell proliferation positively, in this meristem (Bartrina et al., 2011; Vanstraelen and Benková, 2012). The highest cytokinin biosynthesis and reaction location vary in the shoot apical meristem, and these are important for the locating of the WUSCHEL transcription factor expression domain (Chickarmane et al., 2012; Zürcher et al., 2013). Auxin is effective on enhancing the sensitivity of the mitotically less active cells in shoot apical meristem to cytokinin (Schaller et al., 2015).

Cytokinin–auxin ratio is an important signal in creating cell phenotype. Since auxins induce cell division, they are involved in creating meristem in both unorganized tissues or special organs (George et al., 2007). It has been stated in some plants that the balance between cytokinin and auxin is critical for organogenesis and in other plants, not only it is not necessary to use little amounts of auxins but it acts as an inhibitor of cytokinin accumulation. These effects differ depending on plant type. In some cases, combination of cytokinins to each other has resulted in PR increase. Our results on G × N15 rootstock showed that the combined use of cytokinins with auxins caused higher shooting than when using them alone and among the used treatments in the present study, the combination of 1 mg/l BAP with 0.1 mg/l resulted in the highest (10.67) shooting per explant. These results are indicating that the interaction among hormones and also concentration of used hormones are effective on enhancing *in vitro* shooting. So that after combining BAP and IBA, the medium containing 1.5 mg/l KIN along with 0.05 mg/l NAA showed high shooting (9.8). Our results were in accordance with the findings of Hepaksoy and Tanrisever (2004) on sweet cherry, Ruzic and Vujovic (2007) and Dolgov et al. (2010) on *Prunus domestica* that all indicated that the highest shooting rate was obtained in the combination of BAP and IBA treatment. The present study findings are in opposite of Sepahvand et al. (2011) on GF677 vegetative rootstock since they introduced BAP and NAA combination as the most appropriate compound for shooting. Here, the combination of BAP and IBA was the most effective treatment which is in accordance with other researchers’ findings conveyed that the effective shooting depends on the cytokinin type and concentration as well as suitable concentrations of cytokinin–auxin, i.e., using BAP along with low amounts of IBA is favorable for shooting (Sedlák and Paprstein, 2007; Ruzic and Vujovic, 2008). The positive effects of auxin–cytokinin can refer to the auxiliary effects of auxins to cytokinins in cell cycle regulation. Pasternak et al. (2000) indicated that auxin has a role in DNA replication process and cytokinin has a role in the cell cycle regulation. Cytokinins are involved in cell cycle regulation along with auxins. They may induce D type (D_3_) cycles and so the progress of cell cycle from G_1_ to S and may G_2_ to M transfer is achieved by CDC2 gene expression induction by H_1_ kinase histone and de-phosphorylation induction by Cdc25. Our adverse obtained results can be due to the fact that shooting is under influence of many factors such as genotype, culture medium (Molassiotis et al., 2003), hormones, carbohydrates (Ruzic et al., 2003; Nowak et al., 2004), different kinds of hormones (Pruski, 2007; Ruzic and Vujovic, 2008; Sedlak and Paprstein, 2008), different kinds of agar, explant type and different light periods (Magyar-Tábori et al., 2010). Researches have shown that high concentrations of hormonal combinations can decrease LS, quality and rate of shooting and increase callus development as low concentrations auxin can induce cell division but can act as an inhibitor of axillary bud growth in higher concentrations (Bagheri et al., 2009). In accordance with our findings, in some almond varieties like Shahroud 7, increase in BA concentration from 2.5 to 3 mg/l resulted in leaf malformation and verification and also PR increase which caused decreasing LS likely because of shoot competition for medium nutrients uptake (Shekafandeh and Khosh-Khui, 2007; Shekafandeh, 2010). Moreover, we found that CW enhances by increasing auxin concentration. Since callus induction in explant is a role of auxin so, it can be due to that role of auxin and also increasing callus production causes lower shoot quality. High auxin concentrations bring about increasing ethylene production and ethylene accumulation in tissue culture vessels prevents of plant tissues growth and development (George et al., 2007; Yaseen et al., 2009). High concentrations of the combination of cytokinin–auxin resulted in high callus production at the end of new micro-shoots which is in accordance with previous works in which high concentrations of BAP-NAA combination resulted in lower shoot number since callus was produced at the end of proliferating shoots (Phulwaria et al., 2014).

Data analysis of our experiments using ANN-GA modeling and optimization procedure showed that this method can be considered as an efficient method for analyzing *in vitro* growth data in proliferation stage. ANN-GA has been also successfully used for modeling and optimization of improved *in vitro* growth condition estimation for kiwifruit (Gago et al., 2014) which is in agreement with our obtained results.

## Conclusion

The results of analyzing five different cytokinin–auxin hormonal combinations and evaluation using ANN-GA hybrid on *in vitro* growth parameters of G × N15 showed that: (1) 1.2 mg/l BAP + (0.098 mg/l) IBA was found as the best cytokinin–auxin combination for proliferation, (2) the results of verification analysis showed that ANN-GA is an efficient method for predicting an optimizing cytokinin–auxin hormonal combination in *in vitro* proliferation of G × N15.

## Author Contributions

All authors listed have made a substantial, direct and intellectual contribution to the work, and approved it for publication.

## Conflict of Interest Statement

The authors declare that the research was conducted in the absence of any commercial or financial relationships that could be construed as a potential conflict of interest.

## Abbreviations

ANN: artificial neural network
BAP: 6- benzyle amino purine
CW: callus weight
GA: genetic algorithm
IAA: indole – 3- acetic acid
IBA: indole – 3- butyric acid
KIN: kinetin
LS: length of shoot
MS: Murashige and Skoog (1962)
NAA: 1- naphthalene acetic acid
NS: number of shoot
QI: quality index
TDZ: thidiazuron
VSR: variable sensitivity ratio
